# Cognitive Flexibility Moderates the Predictive Effect of Phonological Awareness on Focus Structures in Chinese Preschool Children

**DOI:** 10.3390/brainsci14040324

**Published:** 2024-03-28

**Authors:** Xueqing Tan, Jun Song

**Affiliations:** 1 Faculty of Psychology, Tianjin Normal University, Tianjin 300387, China; tanxq@hpu.edu.cn; 2 School of Mechanical and Power Engineering, Henan Polytechnic University, Jiaozuo 454000, China

**Keywords:** phonological awareness, focus structure, cognitive flexibility, inhibitory control, preschool children

## Abstract

Focus structures, a complex aspect of information structure in language, have garnered significant attention in psycholinguistics. The question of whether Chinese preschoolers aged 4–6 years possess the ability to process focus structures in oral communication, and how cognitive factors influence this ability, remains a research focal point. To address this, we recruited 100 Chinese preschoolers aged 4–6 years as participants in our study. This study manipulated the positions of focus particles in sentences to investigate the impact of phonological awareness on young children’s comprehension of focus structures. Additionally, we examined the mediating roles of cognitive flexibility and inhibitory control. Our findings indicate the following: (1) phonological awareness positively predicted the accuracy of focus structural processing; (2) inhibitory control did not significantly predict the accuracy of focus structural processing; and (3) cognitive flexibility partially mediated the relationship between phonological awareness and focus structural comprehension. These results confirmed the predictive effect of cognitive flexibility on children’s comprehension of focus structures. Moreover, they demonstrate that young children’s phonological awareness can predict their focus structure comprehension ability through the mediating role of cognitive flexibility. This suggests that children’s cognitive flexibility can aid in understanding sentences with focus structures.

## 1. Introduction

Focus is crucial, as it highlights the most significant or emphasized aspect of an utterance [1,2]. There are a number of ways in which linguistic entities can become focused. Focus can be marked prosodically (by a pitch accent) or syntactically [3,4]. If the focus structure of a sentence successfully reflects the intention of the speaker, it necessarily has consequences for how a sentence is processed and perceived by the listener. The cognitive characteristics of preschool children’s focus processing have garnered significant interest in psycholinguistics, yet there remains debate over how preschoolers represent focus structures [5,6,7,8]. Acquiring the ability to process focus structures suggests that children have mastered syntactic recursion and component attachment at a young age and can engage in pragmatic reasoning by integrating contextual information. Numerous studies have utilized focus particles as research material to explore the cognitive characteristics associated with children’s comprehension of focus structures [9,10,11,12]. Therefore, this study focuses on exploring how the cognitive ability of preschool children affects their comprehension of focus structures after hearing them.

### 1.1. Syntactic Analysis of Focus Structures

In spoken language and natural reading, focus particles are often used to mark the focal information of sentences. Additionally, focus particles can be associated with a focused expression, and thus provide a further cue that a focused expression is present in an utterance. The positions of focus particles are different, and the sentence structures are also different, leading to different truth values of sentences [13]. Pre-subject and pre-object focus sentences are two types of sentences in linguistics, distinguished by the placement of words such as “only”. For example, in a pre-subject focus sentence, “only” is before the subject. In a pre-object focus sentence, “only” is before the object, for example, as follows:

(a) Only the cat holds the flag.
(b) The cat only holds the flag.

In these cases, (a) emphasizes the subject “the cat” rather than other components, while (b) emphasizes the object “holds the flag” rather than other components. Studies have shown that children acquire the use of focus particles early in language development. Zhou and Crain’s research [4] demonstrates that children begin to use focus particles at an early age. Additionally, some researchers suggested that the placement of focus particles is flexible and can vary depending on the context and intention of the speaker [2,3,4,8,14]. Overall, while children are capable of using focus particles, they still face challenges in comprehending and manipulating complex focus structures, particularly pre-subject focus sentences [5,6,10,15]. For example, in a picture, a cat holds a flag, a duck holds a flag and a balloon, and a frog holds a balloon. Some children mistakenly believe that sentences like “Only the cat holds the flag” and “The cat only holds the flag” are both correct. This suggests that children have difficulty discerning the correct focus structure and applying it to their understanding of the sentence.

According to the alternative semantic theory, the function of focus goes beyond its semantic value; it also serves as a contrastive alternative with the same semantic type as that of the focus [13]. This means that the appearance of focus particles prompts the consideration of alternative meanings, potentially adding to the complexity of processing focus sentences. In pre-subject focus sentences, not only do we have to compare contrastive alternatives for the object, but we also need to compare contrastive alternatives for the subject. This dual comparison adds to the intricacies of the processing, making it more taxing on cognitive resources. Overall, more research is needed to understand the cognitive mechanisms behind preschoolers’ processing of focus structures and how they develop this ability over time.

### 1.2. Cognitive Factors Affecting the Understanding of Chinese Focus Structures

In recent years, researchers have been paying attention to the cognitive characteristics of focus structures in Chinese preschool children. Tan et al. [16] used a visual world paradigm to investigate the characteristics of focus structures in Chinese preschool children aged 4 to 6 years old. The results showed that 4-year-old children are prone to ignore the existence of focus particles. In their experiment, regardless of whether the children heard the pre-object-only sentence or the pre-subject-only sentence, the children only paid attention to the subject and object of the sentence and did not look at the objects in other areas (object alternatives and subject alternatives). The 5-year-old children showed more difficulty in understanding pre-subject focus sentences, while the 6-year-old children had basically mastered the pre-subject focus sentences [16]. This result confirmed that the age between 5 and 6 years old is important for cognitive development regarding focus structures [11,15]. Nevertheless, the aforementioned research solely examined the eye movement processing characteristics of Chinese children’s comprehension of focus structures, without delving into specific cognitive factors.

Some studies suggested that since children may not have fully mastered syntactic rules, they may be unclear about the scope of focus [5,6], or they may struggle with computing the alternatives in pre-subject and pre-object sentences [15]. However, these studies focused on speculating about potential reasons that impact children’s comprehension of focus structure sentences, without specifically exploring which cognitive abilities influence their understanding. Currently, there are two prominent views in the research on cognitive abilities that affect focus structure. One view is that the pre-object focus structure is the default structure, and when understanding pre-subject sentences, people need to inhibit their cognitive understanding of default sentence structures [17,18]. Therefore, the ability for inhibitory control needs to be involved. Inhibitory control is a subcomponent of executive function. Another perspective is that although children can understand both types of focus structures as quickly as adults, their cognitive flexibility leads to them having difficulty switching between the two types of sentences in the same experimental sequence, leading to interference effects [11]. Cognitive flexibility is another subcomponent of executive function. From a neurocognitive point of view, the cognitive flexibility distinguished between two types of cognitive flexibility: spontaneous flexibility and reactive flexibility [19,20,21]. Spontaneous flexibility refers to the ability to generate a diversity of ideas. Spontaneous flexibility tasks typically require subjects to access various classes and categories of knowledge while by-passing automatic and habitual responses and strategies in order to attend to novel features of knowledge, and its common paradigms include the language association fluency paradigm, which is to create words based on morphological features rather than semantic features, and alternative use testing, which is to choose a random item in daily life, such as a chair, and try to list as many uses of the item as possible within two minutes. In contrast, reactive flexibility refers to the free switching of cognition and behavior according to specific needs and situations. The common paradigm is the task-switching paradigm and the Wisconsin Card Sorting Test [22]. Ibrahim et al. did a series of tests designed to tap two types of cognitive flexibility. They found several significant differences where the balanced bilinguals (Hebrew and English) performed better relative to individuals from the same cultural background. This study focuses on reactive flexibility [22].

Additionally, previous studies have shown that children’s difficulty in understanding language is not only influenced by general cognitive abilities but also by metalinguistic processing skills such as phonological awareness [23,24,25]. The process of oral language and dialogue comprehension in preschool children requires phonological processing. Eviatar and Ibrahim explored the relationship between exposure to two languages (Semitic language) and metalinguistic abilities in kindergarten children. Arabic-speaking children who had been exposed to both to spoken Arabic and literary Arabic (diglossic) were compared to Russian–Hebrew bilinguals and Hebrew monolinguals in tests that included language arbitrariness and phonological awareness. The results showed that Arab children mimicked those of the Russian–Hebrew bilinguals and differed from those of the Hebrew monolinguals. Based on these results they concluded that there is significant effect of the relationship between exposure to two languages and the emergence of metalinguistic skills in childhood [26]. Tummer and Herriman define phonological awareness as the ability to react and manipulate phoneme segments [27]. For example, if asked about the word “bike”, which contains several syllables, the correct response is 2, /bai/ and /k/. If a child is asked to remove the /l/ from the word “sleep” and say what the word is, the correct response is “seep”. Previous studies have shown that preschool children possess phonological awareness skills, but they are not yet fully developed and stable [14,28]. Phonological awareness in children can predict their reading ability, and numerous studies have shown that phonological awareness has a predictive effect on reading-related abilities in early elementary school children [27,29]. The lexical quality hypothesis posits that the phonetics of a word serve as a bridge to its semantics, and the meaning of words can be accessed through verbal language and phonetic matching [30]. The reading systems framework outlines that language processing involves word recognition, semantic extraction, sentence analysis, reasoning, and other comprehension processes [31]. Therefore, phonological awareness serves as a ‘bottom-level’ skill in the reading process, and it involves complex cognitive processes for understanding sentences. This bottom-level skill may be directly or indirectly associated with ‘top-level’ reading comprehension.

Phonological awareness has strong explanatory power in predicting Chinese children’s reading comprehension [32]. The understanding of focus structure sentences is a type of sentence reading comprehension. However, most studies do not account for the influence of phonological awareness on focus structures.

Moreover, previous studies examining the comprehension of focus structures among children aged 4–6 have predominantly utilized sentence–picture judgment tasks [15], truth value judgment tasks [7], and visual world paradigms [10,11]. These studies share a commonality in that the experimental sentence materials are presented auditorily, potentially confounding the influence of individual differences in phonological awareness among children.

### 1.3. Hypotheses

The present study aims to investigate the predictive effects of phonological awareness, inhibitory control, and cognitive flexibility on preschool children’s comprehension of focus structures. Specifically, this study hypothesizes the following:

**Hypothesis H1:**  *Preschool children’s phonological awareness significantly correlates with their comprehension of focus structures.*

**Hypothesis H2:**  *Preschool children’s inhibitory control significantly correlates with their comprehension of focus structures.*

**Hypothesis H3:**  *Preschool children’s cognitive flexibility significantly correlates with their comprehension of focus structures.*

### 1.4. The Relationship between Cognitive Flexibility, Inhibitory Control, and Phonological Awareness in the Understanding of Focused Structures

Numerous studies have demonstrated a correlation between phonological awareness and executive function, such as cognitive flexibility and inhibitory control [33,34,35,36]. In discourse comprehension, cognitive flexibility may support readers in flexibly considering phonetic and semantic information while reading [37,38], and simultaneously considering multiple aspects of a story or situation to facilitate reading comprehension [39], such as indicating whether a letter is a vowel or consonant, if a number is even or odd [37], as well as switching between different tasks. The development of cognitive flexibility begins at the age of 2 and gradually progresses between ages 3 and 5 [40,41,42]. Research has found that the cognitive flexibility of 4- to 5-year-old children can influence their reading comprehension and predict the reading performance of these developing readers [43,44]. Studies have indicated that cognitive flexibility may promote the development of individual phonological awareness, and consequently enhance reading ability [38,45]. However, previous research has mostly focused on stories or short texts, paying less attention to materials with complex syntactic structures. Knudsen et al. [46] suggested that cognitive flexibility significantly predicts children’s accuracy in sentence comprehension. On the other hand, Qi et al. [47] found that the relationship between cognitive flexibility and sentence comprehension speed was significant, whereas the correlation with sentence comprehension accuracy was not; however, their participants were college students. They argued that college students generally have high levels of cognitive flexibility and sentence comprehension accuracy, so even if their cognitive flexibility is relatively high, it is not sufficient to improve their sentence comprehension accuracy. In the case of children, however, relatively high levels of general cognitive flexibility are helpful in enhancing sentence comprehension accuracy. Nevertheless, this explanation has not been experimentally verified further. This study involved 4- to 6-year-old children as participants, as their cognitive flexibility is in a developmental stage, which allows for an exploration of the relationship between cognitive flexibility and accurate comprehension of complex sentence structures. Based on this, we propose Hypothesis 4 in this study: phonological awareness can predict the comprehension of focus structures through cognitive flexibility.

In addition, preschool children need to actively discover salient information during the reading process and focus on information related to the topic, in order to gain an accurate and complete understanding of the reading content. Therefore, children need to suppress irrelevant information that interferes with their ability to obtain complete processing of focused information [48]. Zhao et al. [23] found significant differences in phonological awareness and inhibitory control between Chinese reading-disabled children and control group children. However, after controlling for phonological awareness, the differences between the experimental group and control group in the day–night task and the Stroop task remained significant. They believed that inhibitory control could independently affect the Chinese reading-disabled children’s reading comprehension. Although this study failed to prove whether phonological awareness could predict reading comprehension through inhibitory control, some studies have shown that inhibitory control training can improve their phonological category learning performance [36]. Based on this, we propose Hypothesis H5: phonological awareness can predict the understanding of focus structures through inhibitory control.

**Figure 1:**
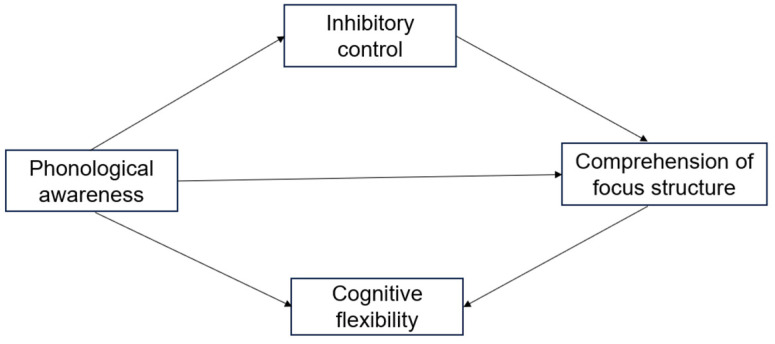
Hypothesis models between variables.

In summary, in Chinese, phonological awareness, cognitive flexibility, and inhibitory control are closely related to the reading comprehension ability of young children. The focus structure is a complex syntactic structure with pragmatic characteristics, making it more difficult to understand and more complex to process. However, previous studies on preschool children’s understanding of focus structures have often overlooked the significance of general cognitive abilities in their comprehension of focus structures during oral conversations. In addition, preschool children have not received formal teaching, and there are still some differences in the development of processing pre-subject-only and pre-object-only focus structures. Given that preschool children are in the critical period of “learning universal grammar”, their cognitive and language abilities develop rapidly during this stage. Investigating their cognitive skills related to understanding focus structures can provide more theoretical and practical guidance for promoting the development of children’s pragmatic abilities. Investigating the cognitive skills related to understanding focus structures can provide more theoretical and practical guidance for promoting the development of children’s pragmatic abilities. According to the hypothesis of vocabulary representation quality, this study intends to incorporate phonological awareness, inhibitory control, and cognitive flexibility into a model to explore the relationship between early phonological awareness and focus structures, as well as whether inhibitory control and cognitive flexibility play a mediating role. We aim to answer three research questions: (1) What is the predictive value of phonological awareness for Chinese preschool children’s understanding of focus structures? (2) Do cognitive flexibility and inhibitory control play a part in the understanding of focus structures? (3) Does phonological awareness mediate via the parallel mediating role of cognitive flexibility and inhibitory control? The hypothetical model is shown in Figure 1.

## 2. Materials and Methods

### 2.1. Participants

This study involved 100 children aged 4–6 years old (52 girls) from a kindergarten in Tianjin. Among them, there were 31 children in their first kindergarten year (M = 52.65 ± 4.18 months, 17 girls), 34 children in their second kindergarten year (M = 59.62 ± 15.50 months, 17 girls), and 35 children in their third kindergarten year (M = 77.34 ± 3.89 months, 18 girls). The participants had normal vision and hearing, were right-handed, and had no language or intellectual disabilities. All of the participants spoke Chinese as their native language. All participants were in good health, had not participated in similar experiments before, and received a small gift after the experiment.

### 2.2. Materials

#### 2.2.1. Measuring Cognitive Flexibility

The test tool in this study is the dimensional change card classification task [49]. The test tool in this study is measuring “Reactive flexibility” [20,21]. The test material consists of 7 groups of cards consisting of different sizes, colors, and shapes. Each group has 4 cards, with shapes including triangles, squares, circles, and rectangles, and colors including blue, yellow, green, and red. One group is used for learning and 6 groups are used for formal testing. During the test, the experimenter presents the participant with a group of cards consisting of a large yellow rectangle, a large green circle, a small green triangle, and a small blue circle. The participant is instructed to pick out 2 cards with the same feature and put together as many as possible. The instructions are as follows: “We are going to play a game. First, you have to observe how I play, and then you have to start playing for yourself. Here are 4 cards with different shapes, colors, and sizes. I want to put 2 cards with the same place together. Look at this small green triangle and large green circle. Because they are both green and have the same color, I will put them together. Here are also small blue circles and large green circles. Because they are both round and have the same shape, I will put them together. I can also put the large yellow rectangle and large green circle together, and the small green triangle and small blue circle together because they are both large and small respectively. Think about it. Is there any other way to put them together? If you think there is not, say ‘no’”. After completing the pairing combination, the participant is required to give an explanation. The correct explanation can be scored, such as “both are long”. Each group of cards can be found in 3 combinations. If the participant finds and correctly explains one combination, they can score 1 point. The scoring range is 0–18 points [49,50].

#### 2.2.2. Measuring Inhibitory Control

The head-toe-knee-shoulder (HTKS) task was developed as a complex, extended version of the head-toe task, which has shown reliability and validity in recent studies with preschool children [51,52]. The HTKS includes 20 test trials. After getting used to two types of verbal commands (e.g., “Touch your head” and “Touch your toe”), children were asked to respond in certain ways. The correct response to “Touch your foot” is for the child to touch their own head; the correct response to “Touch your knee” is for the child to touch their shoulder. Correct responses earn 2 points; incorrect responses earn 0 points; if children make any movements in response to an incorrect response to self-correct, ending with a correct action, they earn 1 point. The score ranges from 0 to 40. The commands are given in a consistent, non-random order. Higher scores indicate higher levels of behavioral regulation.

#### 2.2.3. Measuring Phonological Awareness

This study utilized a syllable deletion task developed by Li et al. [53] to assess participants’ awareness of larger units of syllables. In this task, the experimenter presents 2 to 3 syllables to the participant orally, followed by a request to identify the remaining syllables after deletion of a particular syllable. For instance, if the experimenter says “雪球” (means snowball), the participant is asked to identify what remains after the deletion of “雪” (means snow). The correct answer is “球” (means ball). The test includes a total of 20 questions, and each correct answer is worth one point.

#### 2.2.4. Measuring Pragmatic Competence

**Figure 2:**
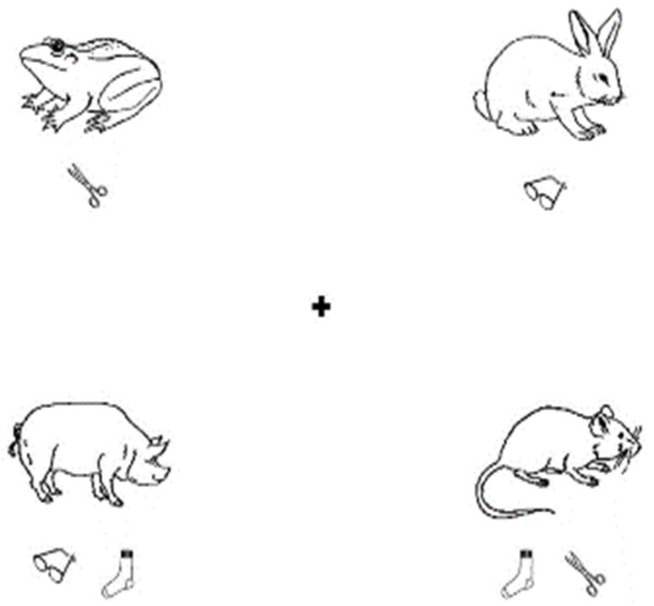
Example of sentence image material.

In the sentence–picture matching task, the experimental material was selected from the Snodgrass and Vanderwart [54] picture library, including 4 simple drawings of animals (mouse, frog, pig, and rabbit), 5 simple drawings of objects, and effective evaluations were conducted on the familiarity and complexity of animals and objects. These animals and objects were freely combined to form 40 pictures, corresponding to 40 experimental sentences. In the experiment, we added 20 filler sentences and pictures, which were randomly distributed together with the experimental sentences. Each picture depicts four animals, with the spatial locations of the animals randomly distributed. One or two items were randomly assigned below each animal (see Figure 2). According to the focus particle “only” in two positions in the sentence, there were two structures corresponding to the sentence material: (1) The pre-subject sentences contain seven words, such as “只有青蛙有眼镜” (means “Only the frogs have glasses”). (2) The pre-object sentences contain six words, such as “青蛙只有眼镜” (means “The frogs only have glasses”). Nevertheless, we controlled for the length of the speech in the recording, there was no difference in the length of the sentence voice presented to the children (*t* = 1.66, *p* = 0.07). There were 20 sentences of each type, with one sentence matching a picture. The proportion of sentence–picture matches and mismatches was 50%. The picture appeared on the computer screen 1s before the onset of the target sentence. Participants responded ‘yes’ (press ‘J’) if they thought the sentence described events in the picture, and responded ‘no’ (press ‘K’) if they thought otherwise. Each trial ended when the participant submitted a response. The participants listened to the sentences, and then made judgments on whether the sentence matched the picture.

### 2.3. Procedure

Participants entered the laboratory and conducted one-on-one tests in the following order: (1) sentence–picture test; (2) phonological awareness test; (3) cognitive flexibility test; (4) HTKS inhibition control test.

## 3. Results

### 3.1. Descriptive Statistics and Correlation Analysis between Variables

The descriptive statistics regarding the variables of interest—inhibitory control, phonological awareness, and cognitive flexibility—are presented in Table 1. Table 2 further details the differences in each variable based on age group. The analysis reveals significant differences in inhibitory control (*F* = 3.13, *η*^2^ = 0.06, *p* = 0.048), phonological awareness (*F* = 26.04, *η*^2^ = 0.43, *p* < 0.001), and cognitive flexibility (*F* = 8.42, *η*^2^ = 0.16, *p* < 0.001) based on age group. These findings suggest that each variable exhibits age-related changes that are consistent with typical developmental trends. However, this study’s primary focus is on the predictive relationships between variables during the preschool period. Therefore, to control for the potential confounding effects of age, a partial correlation analysis was conducted while controlling for age variable(s). The results of this analysis are summarized in Table 2 and indicate the nature of relationships between variables.

Table 2 presents the correlation coefficients between phonological awareness, cognitive flexibility, inhibitory control, and focus structure accuracy. Phonological awareness and cognitive flexibility are significantly correlated with focus structure accuracy, with correlation coefficients of *r* = 0.44 (*p* < 0.001) and *r* = 0.48 (*p* < 0.001), respectively. These findings suggest that higher levels of phonological awareness and cognitive flexibility lead to higher focus structure accuracy. However, the correlation between inhibitory control and pre-subject focus structure accuracy is not significant (*r* = 0.04, *p* = 0.69). These results contribute to our understanding of the relationships between these cognitive processes and focus structure accuracy.

### 3.2. The Predictive Effects of Phonological Awareness and Inhibitory Control on Sentence Comprehension with Different Focus Structures

Using SPSS23.0 and Process v3.5, we conducted a mediation effect test to examine the predictive effects of phonological awareness and inhibitory control on understanding different focus structures. The results are shown in Table 3.

The independent variable had a significant impact on the dependent variable (*β* = 0.33, *p* = 0.004) in the first step of the test, indicating the establishment of the total effect. In the test of model two, the independent variable had a significant impact on the mediating variable (*β* = 0.54, *p* < 0.001). In the third step of the test, the independent variable had a significant impact on the dependent variable (*β* = 0.26, *p* = 0.04), and inhibitory control had no significant impact on the comprehension accuracy of object focus structure sentences (*β* = 0.12, *p* = 0.29). Through the bootstrap technique, the mediating role of cognitive flexibility in the model was tested; it can be seen from Table 4 that the indirect effect value of the comprehension accuracy of pre-object focus structure sentences is 0.04, and the 95% confidence interval [−0.03, 0.1] includes 0, indicating that the indirect effect was not established, and inhibitory control does not play a mediating role in the understanding of pre-object focus structure sentences.

The results of the pre-subject focus sentence comprehension indicate that in the first step of the test, the independent variable has a significant impact on the dependent variable (*β* = 0.34, *p* < 0.001), indicating that the total effect holds. In testing model two, the independent variable was found to have a significant impact on the mediating variable (*β* = 0.54, *p* < 0.001). In the third step of the test, the independent variable had a significant impact on the dependent variable (*β* = 0.43, *p* < 0.001), and inhibitory control had no significant impact on the accuracy of subject focus structure sentences (*β* = −0.13, *p* = 0.17). Through the bootstrap technique, the mediating role of cognitive flexibility in the model was tested, and it can be seen from Table 4 that the indirect effect value of the understanding accuracy of pre-object focus structure sentences is −0.05, and the 95% confidence interval [−0.11, −0.01] does not contain 0, indicating that the indirect effect holds. Inhibitory control plays a masking effect in the understanding of pre-subject focus structure sentences.

### 3.3. The Predictive Effect of Phonological Awareness and Cognitive Flexibility on Sentences with Different Focus Structures

Using SPSS23.0 and Process v3.5 to conduct mediation effect tests, the results are shown in Table 3 and Table 4. When testing the comprehension of sentences with pre-object focus structures, the first step (model 1) showed a significant impact relationship between the independent variable and the dependent variable (*β* = 0.33, *p* = 0.002), indicating that the total effect holds. In the test of model 2, the independent variable was found to have a significant impact relationship with the mediating variable (*β* = 0.31, *p* = 0.003). In the third step of the test, the independent variable had a significant impact relationship with the dependent variable (*β* = 0.27, *p* = 0.04), indicating that the direct effect is significant, while cognitive flexibility has no significant impact on the accuracy of understanding sentences with object focus structures (*β* = 0.18, *p* = 0.53). Through the bootstrap technique, the mediating effect of cognitive flexibility in the model was tested, and it can be seen from Table 4 that the indirect effect value of understanding sentences with pre-object focus structure is 0.05, and the 95% confidence interval [0.06, 0.12] does not contain 0, indicating that the indirect effect holds, indicating that cognitive flexibility plays a partial mediating role in understanding sentences with pre-object focus structures, accounting for 27.96% of the effect.

When testing the comprehension accuracy of pre-subject focus structures, the first step (model 1) showed a significant impact relationship between independent variables and dependent variables (*β* = 0.34, *p* < 0.001), indicating that the total effect holds. In the test of model 2, independent variables had a significant impact on mediating variables (*β* = 0.31, *p* = 0.003). In the third step of the test, independent variables had a significant impact on dependent variables (*β* = 0.29, *p* = 0.004), indicating that the direct effect was significant, and cognitive flexibility had a significant impact on the accuracy of subject focus sentence comprehension (*β* = 0.23, *p* = 0.03). Through the bootstrap technique, the mediating role of cognitive flexibility in the model was tested; it can be seen from Table 4 that the indirect effect value of subject focus structure sentence comprehension accuracy is 0.05, and the 95% confidence interval [0.01, 0.12] does not contain 0, indicating that the indirect effect holds. This indicates that cognitive flexibility plays a partial mediating role in pre-subject focus structure sentence comprehension, accounting for 20% of the effect.

When testing the comprehension accuracy of focus structure sentences (Table 5), in the first step (model 1), the independent variable had a significant impact on the dependent variable (*β* = 0.48, *p* < 0.001), indicating that the total effect was established. In the test of model 2, the independent variable had a significant impact on the mediating variable (*β* = 0.31, *p* = 0.003). In the third step of the test, the independent variable had a significant impact on the dependent variable (*β* = 0.38, *p* < 0.001), indicating that the direct effect was significant, and cognitive flexibility had a significant impact on the correct rate of sentence comprehension for subject focus structures (*β* = 0.33, *p* < 0.001). The mediating effect of cognitive flexibility in the model was tested using bootstrap technology. As shown in Table 6, the indirect effect value of sentence comprehension for all the focus structures was 0.05, and the 95% confidence interval [0.01, 0.09] did not include 0, indicating that the indirect effect was established. This indicates that cognitive flexibility plays a partial mediating role in sentence comprehension for focus structures, accounting for 25% of the effect.

## 4. Discussion

**Figure 3:**
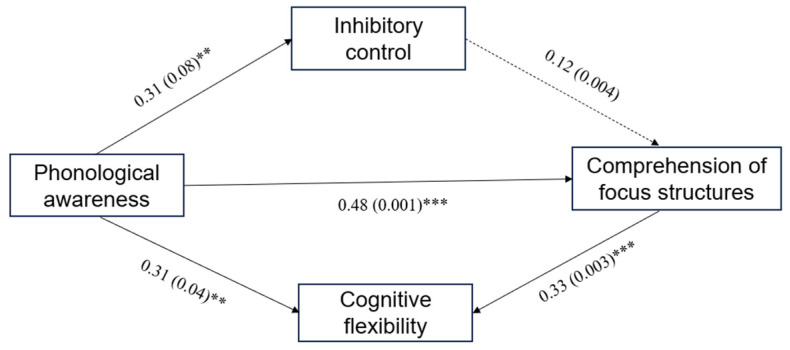
The mediating role model of cognitive flexibility between phonological awareness and pragmatic ability (Note: ** *p* < 0.01, *** *p* < 0.001).

This study is the first to investigate the predictive role of cognitive abilities, such as phonological awareness, inhibitory control, and cognitive flexibility, on the understanding of focus structures in Chinese-speaking children using a sentence–picture matching task (Figure 3). The results showed that there was a significant main effect in age groups on the understanding of both pre-subject and pre-object focus structure sentences. As children aged, the processing of pre-subject and pre-object focus sentences became easier, but the understanding of pre-subject focus sentences became more difficult. Children in different age groups showed different performances in comprehending pre-subject and pre-object focus structures. For children who were 6 years old, the processing of object-related clauses was easier, but there was no difference in the processing difficulty between the two types of clauses for 4-year-old children. At the same time, the cognitive abilities of phonological awareness, inhibitory control, and cognitive flexibility in children aged 4–6 years also improved significantly over time. After controlling for age, phonological awareness and cognitive flexibility still significantly predicted the accuracy of focus sentence understanding. Cognitive flexibility played a partial mediating role in phonological awareness and focus sentence comprehension. In the understanding of pre-object focus sentences, a mediating effect of cognitive flexibility existed and could explain 27.96% of the total effect, while in the understanding of pre-subject focus sentences, a mediating effect of cognitive flexibility existed and could explain 20% of the total effect. However, a mediating effect of inhibitory control in phonological awareness on the comprehension accuracy of focus sentences was not found in this study.

### 4.1. The Predictive Effect of Preschool Children’s Phonological Awareness on Their Understanding of Focus Structures

From the above results, it can be seen that phonological awareness in preschool children has a direct predictive effect on their accuracy in comprehending sentences with focus structures, which is in line with our hypothesis. Phonological awareness has a certain explanatory power for the accuracy and fluency of word decoding. Preschool children with better phonological awareness may be able to better cut and manipulate the continuous flow of speech in oral language and may be more sensitive to the phonological clues in oral vocabulary, thus promoting their understanding of complex focus structures. Four-year-olds are able to perceive the existence of focus [10,16,55]. Children can keenly perceive the existence of focus prominence through subtle phonological changes, which is beneficial for deep understanding of sentences and improving children’s accuracy with focus structures. The results of this study expand the relationship between phonological awareness and reading ability in Chinese, indicating that phonological awareness is a necessary skill for reading, which can not only predict general sentence comprehension [18,19], but also predict the understanding of focus structures.

However, this study falls short of elucidating the causal linkage between phonological awareness and the comprehension of focus structure. Numerous prior investigations have employed prosodic prominence as a marker of focus, yet they have overlooked the role of phonological awareness. Tong et al. have highlighted that prosodic sensitivity anticipates English word recognition via the transfer of segmental phonological awareness between Cantonese and English, with segmental phonological awareness in both Chinese and English serving as a moderator in predicting the relationship between prosodic sensitivity and word recognition [56]. In future research, prosodic prominence could serve as a valuable tool to delve deeper into the experimental exploration of the intricate interplay between phonological awareness and focus perception.

### 4.2. Phonological Awareness Indirectly Affects Preschool Children’s Understanding of Focus Structures through Cognitive Flexibility

Previous studies have reported that cognitive flexibility has a stable predictive effect on reading comprehension scores among 7- to 11-year-olds [38] and on the comprehension speed of different sentence structures among college students [47]. The results of this study are consistent with those of Knudsen et al. [46] and add evidence from preschool children to suggest that cognitive flexibility has an important predictive effect on the development of children at all stages. The results support the impact of cognitive flexibility on young children’s reading comprehension [44] and support the research hypothesis that cognitive flexibility predicts young children’s understanding of focus structures. This study found that phonological awareness has a stable predictive effect on the comprehension accuracy of focus sentences, but cognitive flexibility has different predictive effects on the accuracy of understanding focus structures. When the focus is placed before the object, the mediating effect of cognitive flexibility is higher. This may be because for young children, understanding pre-subject focus sentences is more difficult than understanding pre-object focus sentences [7,10]. Pre-subject focus structure requires young children to flexibly switch between two sentence types, identify the position of focus particles in sentences, and compare and analyze all subject animals and object items in the picture, which requires children to flexibly switch their thinking modes. However, the overall level of cognitive flexibility development among 5- to 6-year-olds is low [56], so in pre-subject focus structure sentences, more cognitive abilities are required, resulting in a smaller mediating effect of cognitive flexibility in pre-subject focus structures than in pre-object focus structures.

Given that there is a clear significant relationship between exposure to more than one language and cognitive flexibility and metacognitive processes (phonological ability) in Semitic languages [21,26], it is extremely important to consider that the applications of this study are available for bilingual children or across languages.

More than 50% of the world’s population speaks two languages or is fluent in both. The concept of the "bilingual advantage" suggests that people who are proficient in two languages may develop cognitive advantages, especially in the field of executive functions. Future research can be further extended to the field of bilingualism to explore reactive flexibility and spontaneous flexibility. But, this study is firmly rooted in Chinese research, taking into account the significant linguistic disparities between Chinese and Semitic languages. Notably, Chinese characters, often pictographic and ideographic, are read horizontally from left to right, standing in contrast to the reading direction of Arabic, a representative of Semitic languages, which proceeds from right to left. Furthermore, Arabic orthography specifically forbids the engagement of the right hemisphere (RH) in letter identification, adding another layer of complexity to the task of reading the language. Moreover, Arabic possesses unique orthographic and linguistic features that may further compound the challenges associated with reading it [57]. Therefore, whether the findings of this study can be extrapolated to Semitic languages remains an open question, necessitating further empirical support.

### 4.3. The Predictive Role of Cognitive Flexibility and Inhibitory Control on Understanding of Focus Structures

Previous studies have placed more emphasis on the predictive role of cognitive flexibility and inhibitory control on reading comprehension. Some scholars have also explored the predictive role of cognitive flexibility in the comprehension of affirmative and negative sentences [47]. Currently, there is little research exploring the predictive role of inhibitory control on focus structure understanding. In this study, we did not find any predictive role of inhibitory control in preschoolers’ understanding of focus structures, which is inconsistent with the findings of previous research [58,59]. Firstly, Peng et al. [59] used the Stroop color–word naming task, and the participants’ accuracy reached a high level. As a result, they chose reaction time as a reliable metric for assessing inhibitory control. However, in this study, we focused on accuracy. Perhaps the children’s individual differences are more likely to be reflected in the speed of inhibition control; further research is needed to verify the specific impact and degree. However, in their study, it was found that the ability of rapid naming was highly correlated with inhibitory control. After controlling for rapid naming, the predictive effect of inhibitory control on reading comprehension was no longer significant. Another key difference was the age of the participants. Peng et al.’s study involved fifth-grade children; the participants in this study were preschoolers, whose inhibitory control abilities are still in a period of rapid development, and there are more unstable unknown factors that can affect them. Strasser and Río [60] found that inhibitory control in children aged 4–7 years had a negative impact on reading comprehension. This was controlled for various factors like school time, age, gender, vocabulary, working memory, theory of mind, attention, reasoning ability, and comprehension monitoring. Thirdly, the alternative semantic theory suggests that the focus triggers a series of focus alternatives, and readers need to inhibit the information of these alternatives in order to complete processing. The study failed to identify the role of inhibitory control due to preschool children’s limited language skills, vocabulary association rules, and understanding of vocabulary breadth and depth [61,62]. This limited ability to generate or consider alternative information related to focus information suggests that the role of inhibitory control is restricted.

This study found that inhibitory control does not significantly predict the comprehension accuracy of focus structure sentences. However, cognitive flexibility does significantly predict the comprehension accuracy of pre-subject focus structure sentences. This may be because cognitive flexibility aids in solving complex tasks, and is beneficial for finding solutions to multiple or variable tasks [63]. Therefore, as task difficulty increases, the role of cognitive flexibility in understanding focus information becomes more prominent. Therefore, comprehending pre-subject focus structure sentences is more challenging, allowing the role of cognitive flexibility to be emphasized. Li et al. [64] examined the developmental characteristics of cognitive flexibility in 3- to 4-year-old Chinese children. The study found that when 3-year-old children are required to switch between two incompatible rules, they experience switching difficulties. Cognitive flexibility is associated with children’s reasoning ability [65]. When children generate semantic alternatives, they do not suppress the generation of these alternatives, but rather compare and reason about the content of these alternatives with the content of pictures. This is a characteristic of cognitive flexibility. Therefore, in this study, we found that cognitive flexibility played a significant role, while inhibitory control did not serve as a mediator. Specifically, cognitive flexibility was instrumental in task switching, whereas inhibitory control was not directly involved in this process. Additionally, Best and Miller [66] posited that cognitive switching is emergent at the age of 3, with a critical period of development between 4 and 5 years old. Consequently, 3-year-old children exhibit lower completion rates on cognitive switching tasks, whereas some 4-year-old children are capable of completing two-dimensional task switching, and 5-year-old children can fully master two-dimensional task switching. This explanation aligns with the accuracy results observed in this study. In particular, children’s lower accuracy in comprehending pre-subject focus sentences is not due to the need to suppress pre-object focus processing, but rather is attributed to the processing and conversion of two sentence structures. Specifically, pre-subject focus demands more complex reasoning and comparison processes, and the conversion process and calculations contribute to lower accuracy. This is likely because the processing of pre-subject focus structures requires greater cognitive resources, thereby compromising children’s accuracy.

### 4.4. Research Significance and Prospects

This study is the first to examine the predictive effects of phonological awareness, cognitive flexibility, and inhibitory control on preschool children’s understanding of focus structures. It offers significant theoretical implications for understanding the development and influencing factors of complex syntactic structures in preschoolers. Furthermore, in educational practice, educators can consider focusing on training preschoolers’ cognitive flexibility and phonological awareness, in order to enhance their accuracy in comprehending complex focus structures and correctly grasp speakers’ implicit meanings, thereby promoting their language development.

However, there are some limitations to this study: Firstly, the comprehension of subject–object focus structures is a complex process that is not solely influenced by phonological awareness and cognitive flexibility, but also by other cognitive abilities. For instance, recent studies have found that individual analogical reasoning [67] and working memory [68] significantly impact sentence comprehension. Future research can further explore the integration of these factors. Additionally, this study utilized the accuracy of focus structure understanding as an outcome measure, but did not account for response time. Recent studies have shown that children with Chinese reading difficulties exhibit deficits in inhibitory control that are reflected in timed inhibitory control response tasks [24], which could potentially impact subject–object focus structure understanding as well. Future research can further consider the impact of additional indicators.

Although age was a controlled variable in this study, we still found developmental differences between the 4- to 6-year-olds. Future research can aim to increase the sample size to investigate the core factors that impact children’s understanding of focus structures across different age groups. By expanding the sample size, we can gain a more comprehensive understanding of how age interacts with other cognitive abilities to influence focus structure comprehension. This will provide valuable insights into the developmental trajectory of focus structure understanding and inform educational practices aimed at enhancing language development in young learners.

Furthermore, previous research has demonstrated that cognitive flexibility encompasses both reactive and spontaneous flexibility [20,21], with the present study primarily concentrating on reactive flexibility. According to Rooth’s alternative semantics theory, focus particles can trigger the generation of semantic alternatives [13]. When the focus appears, whether it can effectively evoke more words semantically related to the focus vocabulary actually reflects the spontaneous flexibility of children. Looking ahead to future research, we can further explore how children’s spontaneous flexibility influences their understanding and processing of focal information through word association tasks.

Given the current research momentum surrounding focus and phonological awareness, there is indeed promising evidence to support such exploration. Of course, in future studies, we will balance bilingual languages [21,26,57,69]. Ge et al. investigated the processing of focus in English sentences with preverbal only by L2 learners whose L1 was either Cantonese or Dutch, compared to native speakers of English. They found that both L2 groups showed delayed eye movements to the alternative of focus, which was different from the native speakers of English. Moreover, Dutch learners of English were even slower than Cantonese learners of English in directing fixations to the alternative of focus. They interpreted the delayed fixation patterns in both L2 groups as evidence of difficulties in integrating multiple interfaces in real time [70]. There is prosodic transfer between L1 and L2. The prosodic transfer hypothesis suggests that sensitivity to Chinese tone promotes sensitivity to English stress, and sensitivity to stress further enhances English word recognition. For example, Chinese belongs to non-alphabetic languages, while English belongs to alphabetic languages. Although they differ in orthography, they share many similarities in phonological structure: both languages use onset as the phonological unit and segment based on onset. Studies have found that children with strong Chinese rhyme awareness have higher abilities in recognizing, analyzing, and manipulating phonological units at the rhyme level, which gives them an advantage in acquiring English phonological awareness and word recognition [69]. Therefore, Chinese segmental phonological awareness predicts English segmental phonological awareness, and English segmental phonological awareness further predicts English reading ability. Based on this, we guess that phonological awareness may facilitate the understanding of focused sentences in the transfer between Chinese and English, but further scientific validation is needed.

## 5. Conclusions

The cognitive skills of preschool children, including phonological awareness, inhibitory control, and cognitive flexibility, demonstrate significant improvement with age. Controlling for age, phonological awareness uniquely contributes to the comprehension of focus structures. Furthermore, cognitive flexibility predicts the accuracy of focus structures, particularly for pre-subject focus sentences. This finding not only confirms the predictive effect of cognitive flexibility on focus structures but also reveals that preschoolers’ phonological awareness predicts their focus structure comprehension through the mediating role of cognitive flexibility. However, inhibitory control does not predict the accuracy of focus structure comprehension. These findings highlight the critical role of cognitive flexibility in children’s comprehension of focus structures in different positions.

## Figures and Tables

**Table 1:** Descriptive statistics of each measurement result (means and standard deviations).

	Inhibitory Control	Phonological Awareness	Cognitive Flexibility	Accuracy
Total	32.80 (8.07)	5.78 (3.29)	12.75 (3.45)	0.59 (0.29)
4 years	31.00 (1.84)	3.00 (0.47)	11.07 (0.54)	0.48 (0.03)
5 years	31.55 (1.56)	5.67 (0.54)	12.58 (0.60)	0.55 (0.04)
6 years	35.49 (0.69)	8.34 (0.30)	14.42 (0.54)	0.72 (0.04)

**Table 2:** Partial correlation analysis between various measurement variables.

	Inhibitory Control	Phonological Awareness	Cognitive Flexibility	Pre-Object	Pre-Subject	Accuracy
Inhibitory Control	1					
Phonological Awareness	0.47 ***	1				
Cognitive Flexibility	0.24 *	0.31 ***	1			
Pre-object	0.22 *	0.29 ***	0.32 ***	1		
Pre-subject	0.04	0.35 ***	0.31 ***	0.23 *	1	
Accuracy	0.31 ***	0.44 ***	0.48 ***	0.72 ***	0.66 ***	1

Note: * *p* < 0.05, *** *p* < 0.001.

**Table 3:** Predictability of phonological awareness and inhibitory control on various sentence patterns.

Style	Step	Dependent	Independent	*R*	*R-sq*	*F*	*β*	*t*
Pre-object	Step1	Object accuracy	Phonological awareness	0.40	0.16	9.35 ***	0.33	2.96 **
	Step2	Cognitive flexibility	Phonological awareness	0.50	0.25	15.62 ***	0.54	5.17 ***
	Step3	Object accuracy	Phonological awareness	0.42	0.17	6.62 ***	0.26	2.12 *
			Inhibitory control				0.12	1.07
Pre-subject	Step1	Subject accuracy	Phonological awareness	0.54	0.29	19.87 ***	0.34	3.56 ***
	Step2	Cognitive flexibility	Phonological awareness	0.50	0.25	15.62 ***	0.54	5.17 ***
	Step3	Subject accuracy	Phonological awareness	0.55	0.31	14.04 ***	0.43	3.81 ***
			Inhibitory control				−0.13	−1.40
Accuracy	Step1	Accuracy	Phonological awareness	0.59	0.34	25.01 ***	0.47	4.87 *
	Step2	Cognitive flexibility	Phonological awareness	0.46	0.21	12.96 ***	0.31	2.88 **
	Step3	Accuracy	Phonological awareness	0.59	0.35	17.29 ***	0.41	3.74 ***
			Inhibitory control				0.12	1.25

Note: * *p* < 0.05, ** *p* < 0.01, *** *p* < 0.001.

**Table 4:** Bootstrap mediation effect test results on inhibitory control.

	Effect	Effect Value	LLCI	ULCI
Pre-object	Total effect	0.19	0.06	0.32
	Direct effect	0.15	0.01	0.30
	Indirect effect	0.04	−0.03	0.01
Pre-subject	Total effect	0.25	0.11	0.39
	Direct effect	0.30	0.00	0.14
	Indirect effect	−0.05	−0.11	−0.01
Total accuracy	Total effect	0.20	0.12	0.28
	Direct effect	0.17	0.08	0.26
	Indirect effect	0.03	−0.01	0.07

**Table 5:** Predictability of phonological awareness and cognitive flexibility on various sentence patterns.

Style	Step	Dependent	Independent	*R*	*R-sq*	*F*	*β*	*t*
Pre-object	Step1	Object accuracy	Phonological awareness	0.40	0.16	3.39 *	0.33	2.96 **
	Step2	Cognitive flexibility	Phonological awareness	0.46	0.21	12.96 ***	0.31	2.88 **
	Step3	Object accuracy	Phonological awareness	0.44	0.19	7.41 ***	0.27	2.38 *
			Cognitive flexibility				0.18	1.77
Pre-subject	Step1	Subject accuracy	Phonological awareness	0.54	0.29	19.87 ***	0.34	3.56 ***
	Step2	Cognitive flexibility	Phonological awareness	0.46	0.21	12.96 ***	0.31	2.88 **
	Step3	Subject accuracy	Phonological awareness	0.58	0.36	15.92 ***	0.29	2.81 **
			Cognitive flexibility				0.23	2.44 *
Accuracy	Step1	Accuracy	Phonological awareness	0.59	0.34	25.01 ***	0.48	4.87 ***
	Step2	Cognitive flexibility	Phonological awareness	0.46	0.21	12.96 ***	0.31	2.88 **
	Step3	Accuracy	Phonological awareness	0.65	0.43	23.51 ***	0.38	3.93 ***
			Cognitive flexibility				0.33	3.72 ***

Note: * *p* < 0.05, ** *p* < 0.01, *** *p* < 0.001.

**Table 6:** Bootstrap mediation effect test results on cognitive flexibility.

	Effect	Effect Value	LLCI	ULCI	Percentage
Pre-object	Total effect	0.19	0.06	0.32	
	Direct effect	0.13	0.05	0.29	72.04%
	Indirect effect	0.05	0.06	0.12	27.96%
Pre-subject	Total effect	0.25	0.11	0.39	
	Direct effect	0.20	0.06	0.34	80%
	Indirect effect	0.05	0.001	0.12	20%
Accuracy	Total effect	0.20	0.12	0.28	
	Direct effect	0.15	0.08	0.24	75%
	Indirect effect	0.05	0.01	0.09	25%

## Data Availability

The datasets analyzed during the current study are available from the corresponding author upon reasonable request. The data are not publicly available due to the demands of parents of preschoolers.
